# Quality of nutrition services in primary health care facilities: Implications for integrating nutrition into the health system in Bangladesh

**DOI:** 10.1371/journal.pone.0178121

**Published:** 2017-05-18

**Authors:** Sk Masum Billah, Kuntal Kumar Saha, Abdullah Nurus Salam Khan, Ashfaqul Haq Chowdhury, Sarah P. Garnett, Shams El Arifeen, Purnima Menon

**Affiliations:** 1 Maternal and child health division, International Centre for Diarrhoeal Disease Research, Bangladesh (icddr,b), Dhaka, Bangladesh; 2 Department of Nutrition for Health and Development, WHO, Geneva, Switzerland; 3 Humphrey School of Public Affairs, University of Minnesota, Minneapolis, Minnesota, United States of America; 4 The Children's Hospital at Westmead Clinical School, University of Sydney, New South Wales, Australia; 5 International Food Policy Research Institute (IFPRI), Washington DC, United States of America; National Institute of Health, ITALY

## Abstract

**Background:**

In 2011, the Bangladesh Government introduced the National Nutrition Services (NNS) by leveraging the existing health infrastructure to deliver nutrition services to pregnant woman and children. This study examined the quality of nutrition services provided during antenatal care (ANC) and management of sick children younger than five years.

**Methods:**

Service delivery quality was assessed across three dimensions; structural readiness, process and outcome. Structural readiness was assessed by observing the presence of equipment, guidelines and register/reporting forms in ANC rooms and consulting areas for sick children at 37 primary healthcare facilities in 12 sub-districts. In addition, the training and knowledge relevant to nutrition service delivery of 95 healthcare providers was determined. The process of nutrition service delivery was assessed by observing 381 ANC visits and 826 sick children consultations. Satisfaction with the service was the outcome and was determined by interviewing 541 mothers/caregivers of sick children.

**Results:**

Structural readiness to provide nutrition services was higher for ANC compared to management of sick children; 73% of ANC rooms had >5 of the 13 essential items while only 13% of the designated areas for management of sick children had >5 of the 13 essential items. One in five (19%) healthcare providers had received nutrition training through the NNS. Delivery of the nutrition services was poor: <30% of women received all four key antenatal nutrition services, 25% of sick children had their weight checked against a growth-chart and <1% had their height measured. Nevertheless, most mothers/caregivers rated their satisfaction of the service above average.

**Conclusions:**

Strengthening the provision of equipment and increasing the coverage of training are imperative to improve nutrition services. Inherent barriers to implementing nutrition services in primary health care, especially high caseloads during the management of sick under-five children, should be considered to identify alternative and appropriate service delivery platforms before nationwide scale up.

## Background

Child undernutrition is estimated to underlie 3.1 million deaths annually, equivalent to 45% of all child mortality in low and middle income countries [1]. In South-Asia, estimates show that 37% of children younger than five years of age (under-five) are stunted and 46% are underweight [2,3]. Over the last two to three decades Bangladesh has achieved a remarkable reduction in child stunting and underweight and the prevalence has decreased by 1.3 and 1.1 percentage points per year respectively [4]. Nevertheless, the rates of stunting and underweight remain high and nutrition specific indicators are poor. For example, only 55% of infants younger than five months were exclusively breastfed and key infant and young child feeding (IYCF) practices were implemented in less than one in four infants aged 6–23 months [5]. Nutrition specific interventions during the antenatal period and in the first two years of life can prevent maternal and child undernutrition in high risk populations [1, 6–8].

The first major nutrition programme implemented by the Government of Bangladesh was the Bangladesh Integrated Nutrition Programme (BINP) (1996 to 2002). The core component of this programme was community based nutrition activities implemented by non-government organisations (NGOs). However, it had minimal effect on reducing child undernutrition at the population level [9]. The National Nutrition Programme (NNP) (2006 to 2011), which adopted components of BINP, had a particular emphasis on maternal and IYCF practices. Nevertheless, similar to BINP the NNP lacked coordination, duplicated services, had limited links with the healthcare system and was expensive to administer [10].

In 2011, the NNS was developed under the new Health, Population Nutrition Sector Development Programme (2011 to 2016) to accelerate progress in reducing the persistently high rates of maternal and child undernutrition. The NNS, in contrast to previous programmes, focuses on leveraging the existing health and family planning infrastructure to deliver the nutrition services to target groups, utilising the window of opportunity to intervene within the first 1000 days of life. Both antenatal care (ANC) and management of sick under-five children were identified as critical health service delivery contacts [10, 11]. In addition to the standard services delivered during these contacts, appropriate nutrition services were to be incorporated.

In order to deliver nutrition interventions during ANC and consultations for the management of sick under-five children the NNS provided three key inputs: i) provision of essential equipment, guidelines and nutrition supplements; ii) structural adjustments by branding the integrated management of childhood illness (IMCI) corners (i.e. a separate area for the management of sick under-five children) in upazila (sub-district) health complexes as IMCI-Nutrition Corners; and iii) provision of basic NNS nutrition training, including training on IYCF practices, to all healthcare providers [11]. The key strategies of the NNS includes growth monitoring and promotion, encouraging behaviour change to improve good nutritional practices, vitamin A supplementation, zinc supplementation during treatment of diarrhoea and iron/folic acid supplementation for pregnant women. The NNS commenced in July 2011 and by March 2014 all of the three key inputs of the NNS were implemented in 39 upazila (personal communication with NNS core team members, February 2014).

However, increased commitment and resources do not necessarily translate into improved outcomes; a number of barriers to successful health programme implementation have been identified including poor quality of service [12]. Hence, against the backdrop of two years of implementation of NNS, this study aims to examine the extent of provision of high quality nutrition services during ANC and management of sick under-five children at primary healthcare facilities in Bangladesh. Findings from this study have the potential to guide critical heath systems planning and strengthen nutrition service delivery at routine health care contacts.

## Materials and methods

### Study setting

The present study is part of a broader operational research by The World Bank with additional support from Transform Nutrition. The aims of the broader evaluation were to i) assess the effectiveness of the delivery of the different components of NNS; and ii) assess whether the various interventions are being delivered to the intended beneficiaries at an adequate quality and coverage [13]. This study was undertaken in primary healthcare facilities in 12 upazila (sub-districts) of Bangladesh. The facilities included were upazila health complexes, union health and family welfare centres (UH&FWC) and community clinics. The upazila health complexes include both inpatient and outpatient facilities and are staffed by medical officers, nurses and family welfare visitors (paramedics with 18 months pre-service training) and serve a population of ~200,000. The UH&FWC are outpatient clinics which are staffed by sub-assistant community medical officers (paramedics with four years of pre-service training) and family welfare visitors (FWV) and serve a population of ~20,000. The community clinics are staffed by community healthcare providers (three months of pre-service training), family welfare assistants and health assistants (domiciliary healthcare provider) and serve a population of 6,000 to 7,000 [14].

For this study, 12 upazila were purposively selected where training and logistics had been reportedly provided by NNS. The selected upazila were from the seven administrative divisions in Bangladesh, in four regions (northeast, northwest, southeast and southwest) to cover major geographic, agricultural and social environments. Assessments were undertaken at 12 upazila health complexes (one from each upazila), 14 UH&FWCs and 12 community clinics. One UH&FWC and one community clinic were randomly selected from each upazila. However, an additional UH&FWC, at each of the two upazila in the Netrokona district (Dhaka Division) were included as the facility utilization was low (<5 cases) on the observation days. The study was conducted between December 2013 and September 2014; data collection occurred in May and June 2014. The Ethical Review Committee (ERC) of the icddr,b approved the study (Protocol Number 14034). Written informed consent was sought from participants before participating in the study.

### Assessment of the quality of nutrition services

Quality of care was assessed using the Donabedian conceptual framework in three dimensions, which were structural readiness, process and outcome based, Table 1 [15]. The framework requires knowledge of a pre-existing relationship between the three dimensions which has been previously demonstrated in assessing the quality of nutrition services in Vietnam [16].

**Table 1:** Adapted Donabedian model to assess the quality of nutrition services / indicators, sample and data collection.

Dimension	Indicators/information	Data collection method	Sample
Structural readiness	Availability of equipment guidelines record keeping register/reporting forms	Observation and completion of a checklist for antenatal care consultation rooms and IMCI^a^ -Nutrition Corner	37 health facilities 11 upazila health complexes 14 union health and family welfare centres 12 community clinics
	Nutrition training and knowledge of healthcare providers	Interview with a structured questionnaire	95 healthcare providers (42 ANC providers; 53 IMCI providers)
Process	Nutritional service delivery during provision of antenatal care and management of sick under-five children	Observation and completion of a checklist	381 pregnant women
			826 sick under-five children
Outcome	Mothers/caregivers’ satisfaction of service	Interview and questionnaire	541 mothers/caregivers of sick under-five children

^a^ IMCI = Integrated management of childhood illness.

#### A. Structural readiness—Attributes of the settings in which care occurs

Structural readiness was assessed by observing the presence of equipment, guidelines, drugs and appropriate record register/reporting forms in ANC consulting rooms and IMCI-Nutrition Corners/areas for the management of sick under-five children, as well as determining the training and knowledge of the healthcare providers. The equipment for ANC was assessed by a checklist developed using WHO guidelines [17] and included the availability of weighing scales, stadiometers, blood pressure monitors, stethoscopes, thermometers, picture cards with maternal danger signs, iron/folate and calcium supplementation. Structural readiness in the IMCI-Nutrition Corner/areas for the management of sick children was assessed using the WHO health facility survey tool for assessing outpatient management of under-five children’s illnesses [18]. The checklist was adapted to be consistent with the Bangladesh IMCI protocol. Equipment included weighing scales, infantometer/ stadiometers, tape measures for measuring mid-upper arm circumferences and growth charts. Guidelines included IMCI chart booklet, IYCF guideline, guidelines for deworming and vitamin A distribution and the basic NNS nutrition training manual.

Readiness was assessed in 37 primary healthcare facilities, which consisted of 11 upazila health complexes, 14 UH&FWC and 12 community clinics. The equipment checklist for one of the selected upazila health complexes was incomplete and the data has not been presented.

Of the 95 healthcare providers observed, 42 were managing ANC on the day of the interview and 53 were managing sick under-five children. Knowledge of healthcare providers was assessed by a questionnaire, adapted from the baseline survey tool of Alive and Thrive used in Bangladesh [19]. The questionnaire included 15 questions in two domains i) ANC counselling and ii) IYCF practices. The mean scores of the two domains were used in the analysis.

#### B. Process—What is done in giving care

The process of service delivery was assessed by direct observation of provision of care during ANC and management of the sick under-five children. In total, 381 pregnant women during their ANC visit and 826 sick under-five children in IMCI-Nutrition Corners were observed, Table 1. An ANC observation checklist was developed for this study based on the contextually adapted activities described in the four-visit ANC model outlined in WHO clinical guidelines [20]. Activities monitored were anthropometry, assessment of anaemia and oedema, vitamin and mineral supplementation, counselling on diet and breastfeeding. The four key ANC nutrition services were defined as weight measured and recorded, iron-folic acid supplement provided, calcium supplement provided and dietary advice given. Activities monitored for the management of sick under-five children included anthropometry (length/height and weight), assessment of feeding practices, provision of nutrition advice, oral rehydration solution and zinc for diarrhoea (for children >6 months) and treatment with anti-helminthic medication (for children >1 year).

#### C. Outcome—The effects of care

The primary outcome was satisfaction of mothers/caregivers of sick under-five children with the service. This was assessed by a face-to-face interview using five questions after they had received the service. The questions were related to: whether or not they felt comfortable asking their healthcare provider questions, satisfaction of advice, the environment, waiting time and ease of travelling to the facility. Reponses were scored on a five point ‘Likert scale’ where 1 = ‘very poor’, 2 = ‘poor’, 3 = ‘average’, 4 = ‘good’ and 5 = ‘excellent’. A total satisfaction score (range: 5 to 25) for each mother/caregiver was calculated. A mother/caregiver was described as having a ‘higher satisfaction’ if her average score was greater than the group mean. The reliability of the questions was assessed by Cronbach’s alpha, which was 0.63 and considered acceptable [21]. Out of 826 observed cases of sick under-five children, 541 mothers/caregivers were interviewed, Table 1. Despite the intensive approach of data collection the timing of consultations did not allow enough time to complete the exit interview after all observations of care. In addition, some mothers/caregivers wanted to leave the facility immediately after consultation with service provider.

### Data collection

All study tools were prepared in English and translated into the local language, Bangla (S1 Appendix). The tools were pre-tested and necessary modifications were made before data collection. Data were collected by three assessment teams over two days in each upazila health complex and one day at each UH&FWC and community clinic during May and June 2014. Each team consisted of two research medical officers and one research assistant. All team members received appropriate training on the data collection tools. Prior to data collection, the study medical officers also received national ANC and IMCI training. Research medical officers observed ANC service and management of sick under-five child during outpatient hours (8am to 2pm), as well as interviewed the healthcare providers. Research assistants assessed the healthcare facility and interviewed the mother/caregivers of sick under-five children. All hard copy data forms were reviewed by the research assistants for completeness, consistency and coding of data. Data were entered into a custom designed database with consistency checks and data range restrictions.

### Statistical analysis

Data were analysed using Stata version 13. Results are shown in descriptive statistics. Mean knowledge score between the ANC and IMCI service providers was compared using t-test statistics. The associations between the outcome variables (quality of provision of nutrition services during ANC, satisfaction of services for sick under-five children among the mothers/caregivers with different) and different explanatory variables (care recipients’ or care providers’ characteristics) were measured using chi-squared test statistics. Test for equality of proportions within the subcategories was conducted to identify the significance of differences in proportions. P-value less than 0.05 was considered significant for all the statistical tests.

## Results

### A. Structural readiness

#### Presence of equipment, guidelines and appropriate record/reporting forms

The structural readiness of the health facilities for nutrition service delivery is shown in Table 2. The majority of ANC consultancy rooms had weighing scales, blood pressure monitors and stethoscopes, approximately two-thirds of the facilities had thermometers and picture cards for maternal danger signs and less than a third had the basic NNS nutrition training manual. Iron-folic acid supplements were available in all facilities except for two community clinics. Calcium supplements were available in half of all facilities. Appropriate record keeping/reporting forms in the ANC consulting rooms were available in ~50% (6/11) of the upazila health complexes, but at higher levels in the UH&FWC (11/14) and community clinics (10/12). One UH&FWC and one community clinic had none of the listed equipment, Table 2.

**Table 2:** Structural readiness of health facilities—Availability of equipment, supplements, guidelines and record keeping registers.

	Upazila health complex, n = 11	Union health & family welfare centres, n = 14	Community clinics, n = 12
**Antenatal care rooms**			
Weighing scales	11	12	12
Stadiometers	5	4	11
Blood pressure monitor	10	10	9
Stethoscope	10	11	12
Tape measure	4	6	5
Thermometer	6	5	11
Picture cards with maternal danger signs	5	8	7
Iron and folic acid supplement	11	14	10
Calcium supplement	7	4	6
Basic NNS nutrition training manual	1	3	2
Record keeping register/reporting forms	6	11	10
Facilities with >5 items of equipment	8	8	11
Facilities with none of the above listed equipment	0	1	0
**IMCI**^a^**-Nutrition Corners/areas for the management of sick children**			
Weighing scales	7	4	5
Stadiometers	6	4	3
Infantometer	4	2	2
Tape measure (mid-upper arm circumference)	4	3	6
Growth charts	7	0	3
IMCI^a^ chart booklet	6	4	2
Guidelines for deworming	2	1	0
Guidelines for vitamin A distribution	4	1	1
Basic NNS nutrition training manual	3	0	1
IYCF^b^ guidelines	2	0	0
Record keeping register/reporting forms	8	6	4
Facilities with >5 items of equipment	4	0	1
Facilities with none of the above listed equipment	1	7	5

^a^ IMCI = Integrated management of childhood illness

^b^ IYCF = Infant and young child feeding

Less than half (43%) of the IMCI-Nutrition corners/areas for management of sick children had weighing scales and approximately one third had infantometers, tape measures and growth monitoring cards. Very few (n<6) IMCI-Nutrition corners/areas for the management of sick children had appropriate guidelines including IMCI booklets and IYCF guidelines. One upazila health complex, seven UH&FWC and five community clinics had none of the essential nutrition equipment/guidelines in the IMCI-Nutrition corner/areas for the management of sick children.

Overall, the availability of equipment, drugs and guidelines was higher for ANC compared to management of sick children. Seventy three percent of ANC consultancy rooms had >5 of the 13 essential items and 13% of the IMCI-Nutrition corners/areas for the management of sick children had >5 of the 13 essential items, Table 2.

#### Nutritional training and knowledge of healthcare providers

One-third of the ANC providers were family welfare visitors and were predominantly female (90%), Table 3. Most of the healthcare providers for managing sick under-five children were sub-assistant community medical officers and were predominately male (60%). While, almost 70% of the healthcare providers had received some nutrition training during their career, only 14% of ANC providers and 23% of healthcare providers who were managing sick under-five children had received the basic NNS nutrition training.

**Table 3:** Characteristics of healthcare providers delivering antenatal care and care of sick under-five children at different health facilities.

	ANC ^a^ providers, n = 42	IMCI ^b^ providers, n = 53
	%	%
Designation		
Medical officer	21.4	13.2
Nurse	11.9	7.5
Family welfare visitor	33.3	0
Sub-assistant community medical officer	14.3	50.9
Community healthcare provider	7.1	22.6
Health assistant	0	5.7
Family welfare assistant	11.9	0
Type of facility		
Upazila Health Complex	57.1	62.3
Union Health & Family Welfare Centre	26.2	9.4
Community clinic	16.7	28.3
Age, years (mean ± SD)	42 ± 11.3	38 ± 11.8
Sex, male	9.5	60.4
Education, completed years of schooling (mean ± SD)	13.5 ± 2.4	13.4 ± 2.2
Nutrition training		
Received basic NNS nutrition training	14.3	22.6
Received any nutrition training	54.8	47.2

^a^ ANC = Antenatal care

^b^ IMCI = Integrated management of childhood illness

The overall knowledge score for healthcare providers delivering ANC nutrition advice was 2.5 ± 0.7, with 5 being the maximum score, Table 4. Over 98% of healthcare providers knew about the necessity to advise on iron/folate supplementation and 93% knew about exclusive breastfeeding. Only 54% of healthcare providers were aware of the need to advise on the importance of initiation of breastfeeding within an hour of giving birth. Knowledge about the need to advise on use of iodised salt and the avoidance of early introduction of fluids other than breast milk were both low (<7%).

**Table 4:** Healthcare provider’s knowledge of antenatal care nutrition and infant and young child feeding practices.

Areas explored for knowledge assessment of healthcare provider	Antenatal care providers, n = 42	IMCI provider, n = 53	All providers, n = 95
During antenatal care provider need to advise on			
Iron and folate supplementation	100.0	96.2	97.9
Use of Iodised salt	2.4	3.8	3.2
Early initiation of breastfeeding	61.9	47.2	53.7
Exclusive breastfeeding up to 6 months of age	92.9	92.5	92.6
Dangers of introducing other fluids in the first 6 months	11.9	1.9	6.3
Knowledge score (scale 0 to 5) mean ± SD	2.7 ± 0.7*	2.4 ± 0.6*	2.5 ± 0.7
IYCF ^a^ and feeding during illness			
Initiation of breastfeeding within 1 hour of birth	100.0	96.2	97.9
Exclusive breastfeeding up to 6 months	100.0	94.3	96.8
Breastfeeding be more frequent if the mother is concerned that the baby is not getting enough milk	42.9	43.4	43.2
Mother of a baby <6 months should not stop breastfeeding if mother becomes ill	88.1	88.7	88.4
A baby should be first fed water or other liquid at the age of 6 month	69.0	73.6	71.6
A baby should be first fed semi solid and other family food at the age of 6 month	69.0	69.8	69.5
Minimum number of times a child aged 6 to 24 months should be fed complementary foods	83.3	92.5	88.4
A child aged 6 to 24 months should eat at least 4 food groups	85.7	83.0	84.2
A child <6 months with diarrhoea should be breastfed more regularly	85.7	90.6	88.4
A child >6 months with diarrhoea requires zinc supplementation	54.8	84.9	71.6
Knowledge score (scale 0 to 10) mean ± SD	7.8 ± 1.5	8.2 ± 1.4	8.0 ± 1.5

* mean knowledge score for nutrition during ANC was significantly different (P<0.05) between antenatal care providers and IMCI providers

^a^ IYCF = infant and young child feeding

The overall knowledge score for IYCF practices during illness was 8.0± 1.5, with 10 being the maximum score, Table 4. Almost 98% of the healthcare providers knew that initiation of breastfeeding should commence within one hour of giving birth, 97% knew about the importance of exclusive breastfeeding, 88% knew about continuation of breastfeeding during mother’s illness. Knowledge about age appropriate initiation time of complementary feeding, frequency and diversity of complementary feeding ranged from 70% to 88% of healthcare providers. However, only 43% of healthcare providers knew about the importance of more frequent feeds if the mother was concerned that the baby was not getting enough breast milk.

### B. Process evaluation

Assessment of the process of nutritional service delivery was determined by observing 381 ANC visits and 826 sick under-five children consultations.

#### Assessment of nutritional services during ANC

**Fig 1:**
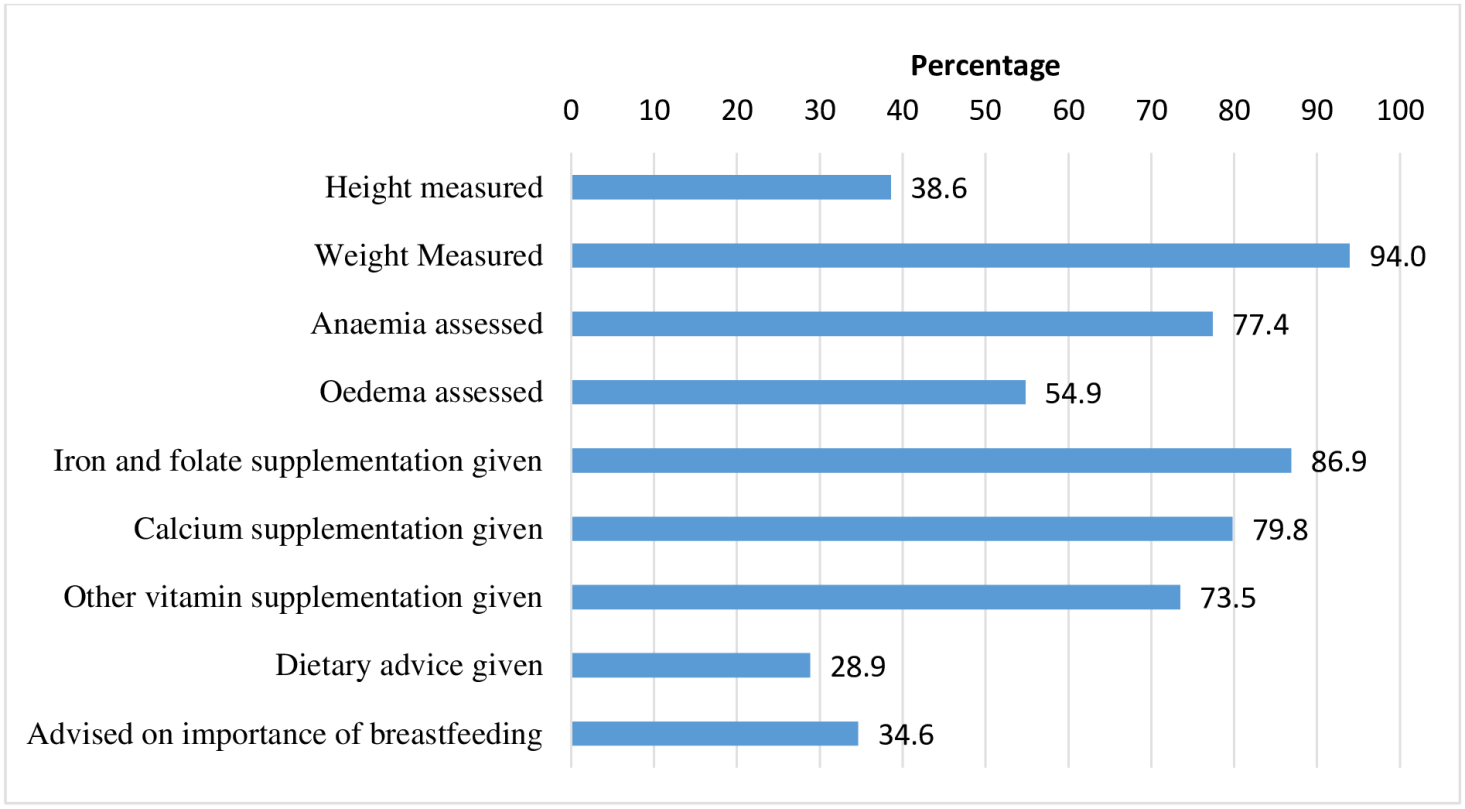
Proportion of women receiving different nutrition services as observed during service provision of antenatal care (n = 381).

The majority of the 381 women who were observed during ANC were younger than 25 years of age (59%), presented later than 13 weeks gestation (>70%) and came for their first ANC visit (51%). The majority (73% to 94%) of women were weighed, had their iron levels assessed for anaemia, and were given iron-folic acid, calcium or other vitamin supplements (Fig 1). Approximately one third of women had their height measured, received dietary advice or were counselled on the importance of breastfeeding. However, only 28% of women received all of the four key ANC nutrition services, i.e. had their weight recorded, were given iron-folic acid supplements, calcium supplements and dietary advice, Table 5. Significantly more (p<0.05) women (>38%) who presented in their second trimester or third trimester (26%) received the four key ANC nutritional services compared to those who presented in their first trimester (10%). Provision of the four key ANC nutritional services was also significantly higher (p<0.001) when medical doctors or nurses were the service provider (62.9%) compared with family welfare visitors or sub-assistant community medical officer (3.3) or community health care provider (2.4%). The median duration of an ANC consultation was 5 minutes [interquartile range 2 to 60], S1 Table. The duration of the consultation was not significantly associated with the designation of healthcare provider or nutrition training.

**Table 5:** Characteristics of women who received the four key nutritional services during antenatal care.

Characteristics	Women eligible for the service, n	Percentage of women who received the four key antenatal nutritional services ^a^, %
**Total**	381	28.1
Mother’s age (years)		
15–19	85	29.4
20–24	138	24.6
25–29	111	29.7
30 and above	47	31.9
Gestational Age		
1st trimester (1–13 weeks)	39	10.3
2nd trimester (14–26 weeks)*	154	37.7
3rd trimester (27 or more)*	135	25.9
Do not know/cannot remember	53	18.9
Order of current ANC visit ^b^		
1st visit	196	25.5
2nd or more visits	183	31.2
Designation of healthcare provider		
Medical Officer or Nurse**	159	62.9
Family welfare visitor or Sub-assistant community medical officer	180	3.3
Community healthcare provider or Family welfare assistant	42	2.4

^a^ Four key antenatal nutritional services were weight recorded, provided iron-folic supplement, calcium supplements and dietary advices;

^b^ 2 missing values

* p<0.05,

** p<0.001 (for equality of proportions test)

#### Assessment of nutritional services during management of sick under-five children

About half (52%) of the 826 children who were observed during clinical management were <2 years of age. Delivery of nutritional services during this healthcare contact was poor. Only one in five children were weighed, <1% had their length/height measured and ~30% were given oral rehydration solution and zinc if diarrhoea was reported, Table 6. Less than one in 10 mothers/caregivers was given advice on feeding and appropriate management of diarrhoea. Median duration of consultation for management of a sick under-five child was 3 minutes [inter quartile range 1 to 18] (S1 Table) and was not significantly associated with the designation of the healthcare provider or nutrition training.

**Table 6:** Assessment of nutritional services during management of sick under-five children as observed during service provision.

Nutritional services during management of sick under-five children	Children of appropriate age eligible for the service, n	Children assessed, %
**Sick under-five children**		
Weight measured and recorded	826	19.7
Weight measured and checked against a growth chart	163	23.9
Length/height measured and recorded	826	0.5
Feeding practices assessed for children <2 years	426	6.1
Children >1 year given an anti-helminthic	527	13.1
Children > 6 months with diarrhoea provided with oral rehydration solution and zinc	194	35.1
**Mother/caregivers advised on**		
Feeding and care	826	6.4
Frequency of feeding	826	6.7
How to give extra liquid/breastmilk & continue feeding during diarrhoea	194	8.2

### C. Outcomes of the nutritional service delivery

Mothers’/caregivers’ level of satisfaction with the nutrition service received for their sick under-five child was assessed to measure the outcome of nutritional service delivery. Level of satisfaction of the service was determined by interviewing 541 mothers/caregivers. Overall, the mean level of service was rated between ‘average’ and ‘good’ for all five indicators measured i.e. whether or not they felt comfortable with their healthcare provider questions, satisfaction of advice, the environment, waiting time and ease of travelling to the facility. Indicator specific data showed that “waiting time to visit a healthcare provider in the facility” had the lowest mean of satisfaction score (S2 Table).

Overall, 61% of the mother/caregivers of sick under-five children had a satisfaction level above the group mean (17.8 ± 2.9) of the total satisfaction score, Table 7. About 66% of the mother/caregivers who had consultation period longer than 3 minutes (median duration of consultation) had a satisfaction score above mean compared to 56% who had shorter consultation duration and the difference was statistically significant (p<0.05). The mothers/caregivers were more likely to have a satisfaction score above mean if their healthcare providers had either IMCI training or basic NNS nutrition training (73.5%) than who had a consultation with providers without any of these training (35%) (p<0.001).

**Table 7:** Characteristics of mothers/caregivers of sick under-five children having satisfaction above the group mean score.

Characteristics	Total, n	Proportion above group mean score, %
Total	541	60.4
Sex of the child		
Male	301	63.8
Female	240	56.3
Duration of consultation*		
≤3 minutes (median duration)	320	56.9
> 3 minutes	221	65.6
Designation of healthcare provider		
Medical Officer or Nurse	30	70.0
Family welfare visitor or Sub-assistant community medical officer	427	61.6
Community healthcare provider or Family welfare assistant	84	51.2
Training status**		
IMCI or basic NNS nutrition training	358	73.5
Neither IMCI or basic NNS nutrition training	183	35.0

* Statistical differences between those who had a higher satisfaction level compared to those with a lower satisfaction is significant with p value < 0.05;

** p value < 0.001

## Discussion

The Bangladesh NNS was developed to bring nutrition into the mainstream healthcare system and accelerate the decrease in the high rates of maternal and child undernutrition. The results from this study indicate that two years after implementation the capacity for nutrition service provision in the healthcare facilities and the process of nutrition services during ANC and management of sick under-five children was poor. The NNS had not been implemented as intended. Nevertheless, most mothers/caregivers rated their satisfaction of the care of their sick under-five child above average.

Overall facility preparedness and service delivery were better for ANC compared to the management of sick under-five children. While the difference was evident at all tiers of healthcare, it was more pronounced for local IMCI-nutrition corners (UH&FWCs and CCs), compared to the sub-district (upazila) level. This could be due to prioritisation of the upazila IMCI-nutrition corners during the implementation of NNS. There was greater availability of essential equipment and pregnant women received a number of essential nutrition services, for example the majority (>80%) were weighed, provided with iron/folate and/or calcium supplements. Women who presented in the second or third trimester were more likely to receive the four key nutritional services than those in first trimester. Late presentation and differential nutrition services by gestational age could be a result of the perception of some healthcare providers. A recent study in Bangladesh, on iron-folate supplementation during pregnancy, indicated that healthcare providers were reluctant to reach out to pregnant women before the second trimester and they had unfounded concerns about supplementation in early pregnancy [22]. Appropriate skills-based training, ensuring that the healthcare providers follow standard guidelines could overcome this gap.

Overall, the assessment of management of sick-child care suggests that there were critical missed opportunities for basic nutrition assessments and counselling. Lack of essential equipment including scales, stadiometers and growth monitoring cards prevented appropriate nutritional assessment and screening for undernutrition. Indeed, <1% of sick children in this study were weighed and had their weight plotted on a growth chart. Without nutritional assessment it is unlikely that there would be appropriate management of undernutrition and appropriate treatment for other childhood illness [23]. Provision of appropriate equipment, improving the coverage and emphasising nutrition services as part of ongoing training may strengthen this activity. However, it is interesting to note that the current IMCI guidelines in Bangladesh already incorporated nutritional services, including methods for assessing a child’s nutrition and feeding status [24]. If these guidelines were implemented as intended, nutrition would already be ‘mainstreamed’ into existing health and family planning infrastructure. It’s not clear why protocols are not been adhered to. Healthcare provider’s perceptions is considered critical in the credibility and acceptability of clinical guidelines [25]. Provision of tailored nutrition training with supportive supervision from management may improve perception and overall adherence to the guidelines [26–30].

There are other challenges relating to the delivery of preventative IYCF messages during a clinical contact, including length of appointment and age of child presenting. Quantitative data indicated that half of the consultations for management of sick children were ≤3 minutes and over 40% of the children were older than two years of age. Short consultations, predominantly as a result of heavy caseloads, can lead to prioritisation of the immediate clinical concern rather than preventative IYCF messages. Alternative healthcare platforms will need to be considered to disseminate information about IYCF practices such as well-child clinics at community level healthcare facilities.

The coverage of basic NNS nutrition training was reported to be low (<25%). Nevertheless, 51% of healthcare providers reported that they had received nutrition training from other governmental and NGO programmes. It is not clear if the low numbers reporting basic NNS nutrition training is a true reflection of the training or a lack of awareness of who is providing the training. Identifying the sources of nutrition training and the extent of the training can be difficult. We also report that ANC providers had better knowledge of ANC than the IMCI providers, even though the healthcare providers in both UH&FWC and community clinics frequently have to cover both ANC and management of childhood illness due to staff shortages [31]. This suggests the need for all healthcare providers to have good knowledge of the nutrition services to be delivered in an equally efficient manner at both contact opportunities.

It is to elucidate why the level of preparedness differs between ANC and IMCI-nutrition corners. Years of schooling and nutrition training, albeit from different sources was similar for the healthcare providers of ANC and IMCI. However, more ANC providers (66%) were qualified as medical officers, nurses or family welfare visitor compared to IMCI providers (20%). In addition to having better qualifications, we speculate that the healthcare centres were better prepared for ANC as ANC has been part of the health services implementation well before the more recently introduced implementation of the IMCI-nutrition corners as part of the NNS. Antenatal care is also fundamentally preventive in nature, rather than curative, thus making the platform itself conducive to further strengthening and integration of nutrition screening and counselling. From a logistics preparedness perspective, the only item in the NNS logistics list exclusive to ANC was the basic nutrition training manual, which had poor availability at all healthcare tiers. In contrast, the materials and tools needed to deliver the nutrition services within the IMCI included several new inputs including growth charts, tape measures for measuring mid-upper arm circumferences and the basic nutrition training manual.

We found the majority of mothers/caregivers of sick under-five children had a satisfaction level above average for the service received, despite the low quality of nutritional services. Expectations of mothers/caregiver may be low and do not always align with the best practice [32]. Consistent with the findings from another study in Bangladesh [33], waiting time was the key reason for dissatisfaction. Higher caseloads at upazila health complexes compared to the UH&FWC [23] might have resulted in longer waiting time, driving the dissatisfaction. Another factor that led to improved satisfaction of the nutrition services during management of sick under-five children included longer consultations. Similar finding has been previously reported [34, 35].

This study had limitations. First, we did not examine satellite or home visits by family welfare assistants and health assistants as it was beyond the scope of the study. Previous qualitative interviews with these staff indicated an almost complete lack of awareness and knowledge about nutrition related services and low exposure to basic NNS nutrition training [13]. Second, the study was conducted in facilities where the NNS had been reportedly introduced. A comparison with facilities where the NNS had not been implemented may have provided a better understating of the quality of nutrition services. In addition, direct observation of service delivery may have encouraged healthcare providers to offer a more thorough service. We anticipate little impact on our findings as both the structural readiness and process of the NNS were low. However, significant strengths of this study were that the assessment covered health care facilities in the major geographic, ethic and socio-cultural regions of Bangladesh and that a combination of methods, questionnaires, interviews and direct observation, were used to assess quality.

## Conclusion

The aim of the NNS, integrating nutrition services into the routine healthcare service delivery contacts, was an ambitious objective. Improving the logistics of providing appropriate equipment and the coverage of training may improve nutrition service delivery. However, there are inherent barriers to implementing nutrition services through the health system including high caseloads, resulting in shorter consultations during ANC and management of sick under-five children. Gestational age specific nutritional counselling package for ANC visits can maximise the efficiency. Missed opportunities to identify undernourished children and lack of time to provide preventive counselling messages can be overcome by introducing well-child clinics in the community or prioritizing home based nutritional screening and counselling by domiciliary workers. Alternative service delivery modes need to be considered including NGOs and other development partners who have demonstrated commitment to nutrition.

## Supporting information

S1 Appendix Structured questionnaire. (PDF)

S1 Table Median consultation time and interquartile range (IQR) of ANC and sick under 5 children within the sub-categories of provider types and their training status. (DOCX)

S2 Table Mothers’/Caregivers’ level of satisfaction with the nutrition service received for their sick under-five child. (DOCX)
