# Supplementary material for: Quality of nutrition services in primary health care facilities: Implications for integrating nutrition into the health system in Bangladesh
Source: PLoS One. 2017 May 18;12(5):e0178121. doi: 10.1371/journal.pone.0178121 (PMC5436890; doi:10.1371/journal.pone.0178121)
Supplement: S1 Appendix — (PDF) [file pone.0178121.s001.pdf]

S1 Appendix: Structured questionnaire

International Centre for Diarrhoeal Disease Research, Bangladesh (icddr,b)

Assessing the progress of the National Nutrition Services (NNS) Program

ANC Observation Checklist

Surveyor Name and ID: ___________________|____|____| Date: ____/____/_______

Observation **Starting time**: |____|____| HH:MM (24 hours)

Observation **End time** (*fill up at the end*): |____|____| HH:MM (24 hours)

District: ______________|___|___| Upazila: _____________|___|___|

Union: _____________________|___|___| Ward No. |_____|

Facility Name/Code: ____|___|___|

Type of facility	Upazila Health Complex..1
	Union Health and Family Welfare Centre..............2
	Community Clinic..3
**Information on Service Provider**	
Designation of health service provider	Medical officer 1
	Nurse 2
	FWV 3
	SACMO 4
	CHCP 5

IMCI Trained: ☐ Yes ☐ No

EmOC Trained: ☐ Yes ☐ No

Basic Nutrition Trained: ☐ Yes ☐ No

Information on Pregnant Woman

No. of ANC visit:

Age of pregnant mother (in years): |___|___| year |______|

Gestational Age (in weeks): |___|___|

weeks LMP: ____/____/_____ EDD: ____/____/_____

Carefully observe the ANC check-up session conducted by the provider and put a (√) in the applicable box.

No.	Observation Points	Responses		
1.	Does the health worker **greet** the woman with respect and dignity?	☐ Yes	☐ No	☐ NA
2.	Does the health worker **explain** to the woman on what the health worker is going to do during ANC?	☐ Yes	☐ No	☐ NA
3.	Does the health worker ask about **any previous ANC visit** at this facility/service provider for current pregnancy?	☐ Yes	☐ No	☐ NA
4.	Does the health worker ask the woman regarding the duration of pregnancy (**Gestational age**)?	☐ Yes	☐ No	☐ NA
5.	Does the health worker ask the woman regarding **LMP** of mother?	☐ Yes	☐ No	☐ NA
6.	Does the health worker ask the woman regarding **EDD** of mother?	☐ Yes	☐ No	☐ NA
7.	Does the health worker ask the woman regarding **last pregnancy/ deliver**y?	☐ Yes	☐ No	☐ NA
8.	Does the health worker ask the woman regarding **history of illnesses**<br>e.g. allergy, hypertension, diabetes, asthma, STI, UTI, heart disease, goiter etc?	☐ Yes	☐ No	☐ NA
9.	Does the health worker ask the woman regarding **history of any medicine use**<br>e.g. iron, folic acid, vitamin, calcium, anti-helminths, anti-hypertensive etc?	☐ Yes	☐ No	☐ NA
10.	Does the health worker, or another staff, weigh and record the **WEIGHT** of the woman today?	☐ Yes	☐ No	☐ NA
11.	Does the health worker, or another staff, measured and record the **HEIGHT** of the woman today?	☐ Yes	☐ No	☐ NA
12.	Does the health worker, or another staff, felt the **PULSE** of the woman today?	☐ Yes	☐ No	☐ NA
13.	Does the health worker, or another staff, checked the **BLOOD PRESSURE** of the woman today?	☐ Yes	☐ No	☐ NA
14.	Does the health worker, or another staff, examined **ANAEMIA** in eyes (Lower palpebral fissure) of the woman today?	☐ Yes	☐ No	☐ NA
15.	Does the health worker, or another staff, examined **JAUNDICE** (in eyes and palm) of the woman today?	☐ Yes	☐ No	☐ NA

No.	Observation Points	Responses		
16.	Does the health worker, or another staff, examined **EDEMA** in leg (in medial malleolus) of the woman today?	☐ Yes	☐ No	☐ NA
17.	Does the health worker, or another staff, examined **ABDOMEN** (e.g. for fundal height) of the woman today?	☐ Yes	☐ No	☐ NA
18.	Does the health worker, or another staff, examined FETAL position, movement, heart rate today?	☐ Yes	☐ No	☐ NA
19.	Is blood test facility available in the health facility?	☐ Yes	☐ No	☐ NA
20.	Does the health worker, or another staff, **Conducted BLOOD TEST**	☐ Yes	☐ No	☐ NA
21.	Is urine test facility available in the health facility?	☐ Yes	☐ No	☐ NA
22.	Does the health worker, or another staff, **Conducted URINE TEST** of the woman today?	☐ Yes	☐ No	☐ NA
23.	Is ULTRASONOGRAM available in the health facility?	☐ Yes	☐ No	☐ NA
24.	Does the health worker, or another staff, **conducted ULTRASONOGRAM** of the woman today?	☐ Yes	☐ No	☐ NA
25.	What **ADVICE (S)** does the health worker provide to the women today?<br>*put a (√) in the applicable box*			
A.	To take more food	☐ Yes	☐ No	☐ NA
B.	To take balanced diet	☐ Yes	☐ No	☐ NA
C.	To take seasonal/available fruits	☐ Yes	☐ No	☐ NA
D.	To take green/coloured vegetables	☐ Yes	☐ No	☐ NA
E.	To drink more water	☐ Yes	☐ No	☐ NA
F.	To take iodized salt	☐ Yes	☐ No	☐ NA
G.	To take rest at least for 2 hours at day time	☐ Yes	☐ No	☐ NA
H.	To maintain personal hygiene	☐ Yes	☐ No	☐ NA
I.	To avoid heavy work	☐ Yes	☐ No	☐ NA
J.	To avoid coitus in first and last trimester	☐ Yes	☐ No	☐ NA
K.	Told about danger signs of pregnancy			
	i. Vaginal bleeding	☐ Yes	☐ No	☐ NA
	ii. Convulsion	☐ Yes	☐ No	☐ NA
	iii. Severe Headache	☐ Yes	☐ No	☐ NA
	iv. Severe Anaemia	☐ Yes	☐ No	☐ NA
	v. Blurring of vision	☐ Yes	☐ No	☐ NA
	vi. Swelling of hands-feet-face	☐ Yes	☐ No	☐ NA
	vii. High blood pressure.	☐ Yes	☐ No	☐ NA
	viii. Excessive fever	☐ Yes	☐ No	☐ NA
	ix. Diminished movement of fetus	☐ Yes	☐ No	☐ NA
	x. Foul smelling vaginal discharge	☐ Yes	☐ No	☐ NA
L.	Told about the importance of going to the **health centre** for complications	☐ Yes	☐ No	☐ NA
M.	Told about importance of **arranging vehicle** ready before delivery	☐ Yes	☐ No	☐ NA

No.	Observation Points	Responses		
N.	Told to save or **save/deposit** money for emergency	☐ Yes	☐ No	☐ NA
O.	Told to do **blood grouping** of the woman	☐ Yes	☐ No	☐ NA
P.	Told to identify **blood donor**	☐ Yes	☐ No	☐ NA
Q.	Told to take **TT vaccine**	☐ Yes	☐ No	☐ NA
R.	Told to visit for **regular antenatal check-ups (ANC)**	☐ Yes	☐ No	☐ NA
S.	Told to take routine **iron and folic acid(IFA)**	☐ Yes	☐ No	☐ NA
T.	Told to use **safe delivery kit**	☐ Yes	☐ No	☐ NA
U.	Told about the use of **delivery mat**	☐ Yes	☐ No	☐ NA
V.	Told about availability/use of maternal **health voucher**	☐ Yes	☐ No	☐ NA
W.	Told about the importance of **breastfeeding**	☐ Yes	☐ No	☐ NA
X.	Told about essential newborn care preparedness **(ENC)**	☐ Yes	☐ No	☐ NA
	i. Drying and wrapping of the newborn	☐ Yes	☐ No	☐ NA
	ii. Initiation of breastfeeding within one hour	☐ Yes	☐ No	☐ NA
	iii. Delayed bathing	☐ Yes	☐ No	☐ NA
	iv. Care of umbilical cord	☐ Yes	☐ No	☐ NA
	v. Extra care for low birth weight baby (LBW).	☐ Yes	☐ No	☐ NA
	vi. Immunization	☐ Yes	☐ No	☐ NA
Y.	Told about the **danger signs of neonate**	☐ Yes	☐ No	☐ NA
26.	Provided a **Pictorial card** with danger signs of pregnancy.	☐ Yes	☐ No	☐ NA
27.	Provided Iron-Folate tablet (IFA)	☐ Yes	☐ No	☐ NA
28.	Provided Calcium tablet	☐ Yes	☐ No	☐ NA
29.	Provided Vitamins	☐ Yes	☐ No	☐ NA
30.	Provided Misoprostol	☐ Yes	☐ No	☐ NA
31.	Does the health worker explain when to return for a follow-up visit?	☐ Yes	☐ No	☐ NA
32.	Does the health worker asks the women if she has any questions?	☐ Yes	☐ No	☐ NA
33.	Does the health worker thank the women after the ANC session?	☐ Yes	☐ No	☐ NA

*Please review the entire observation checklist for any **missing points,***

Fill up with (√) in appropriate box and finish the observation.

International Centre for Diarrhoeal Disease Research, Bangladesh (icddr,b)

Assessing the progress of the National Nutrition Services (NNS) Program

Sick Under-Five Illness Management Observation Checklist

Surveyor Name and ID: ______________________|____|____| Date: ____/____/_______

Observation **Starting time**: |____|____| HH:MM (24 hours)

Observation **End time** (*fill up at the end*): |____|____| HH:MM (24 hours)

District: _____________________|___|___| Upazila: ____|___|___|

Union: _____________________|___|___| Ward No. |___|

Facility Name/Code____|___|___|

Type of facility

Upazila Health Complex..1

Union Health and Family Welfare Centre..............2

Community Clinic..3

Information on Service Provider

Code of the service provider: |____|____|____|

Designation of health service provider

Medical officer 1
Nurse 2
FWV...3
SACMO 4
CHCP..5

IMCI Trained: ☐ Yes ☐ No

Basic Nutrition Trained: ☐ Yes ☐ No

Information on the under-five child

Age of child: |___|___| weeks

Sex of child: ☐ Male ☐ Female

Carefully observe the IMCI session conducted by the provider and put a (√) in the applicable box.

No	Observation points	Responses		
1.	Does the health worker have any IMCI register book?	☐ Yes	☐ No	☐ NA
2.	Does the provider use the IMCI case management sheet in the register book?	☐ Yes	☐ No	☐ NA
3.	Does the health worker, or another staff, weigh and record the WEIGHT of the child today?	☐ Yes	☐ No	☐ NA
4.	Does the health worker, or another staff, measure height/ length and record the height/length of the child today?	☐ Yes	☐ No	☐ NA
5.	Does the health worker, or another staff, check the TEMPERATURE of the child? M P	☐ Yes	☐ No	☐ NA
6.	What REASONS does the caretaker give for bringing the child to the health facility? *(Multiple response possible)*			
	i. Diarrhoea/vomiting	☐ Yes	☐ No	☐ NA
	ii. Fever/malaria	☐ Yes	☐ No	☐ NA
	iii. Fast/difficult breathing/cough/pneumonia	☐ Yes	☐ No	☐ NA
	iv. Ear problem	☐ Yes	☐ No	☐ NA
	v. Well-child visit	☐ Yes	☐ No	☐ NA
	vi. Other(*specify*)____________________________	☐ Yes	☐ No	☐ NA
	vii. None given	☐ Yes	☐ No	☐ NA
7.	Does health worker ask whether the child is ABLE TO DRINK ORBREASTFEED?	☐ Yes	☐ No	☐ NA
8.	Does health worker ask whether the child VOMITS EVERYTHING?	☐ Yes	☐ No	☐ NA
9.	Does health worker ask whether the child has CONVULSIONS?	☐ Yes	☐ No	☐ NA
10.	Is the child VISIBLY AWAKE (e.g., playing, smiling, crying with energy)?	☐ Yes	☐ No	☐ NA
11.	If the child is not visibly awake, does health worker check for LETHARGY or UNCONSCIOUSNESS (try to wake up the child)?	☐ Yes	☐ No	☐ NA
12.	Does health worker ask for COUGH OR DIFFICULT BREATHING?	☐ Yes	☐ No	☐ NA
13.	Does health worker ask for DIARRHOEA?	☐ Yes	☐ No	☐ NA
14.	Does health worker ask/feel for FEVER (or refer to temperature if taken previously)?	☐ Yes	☐ No	☐ NA

No	Observation points	Responses		
15.	Does health worker ask for EAR PROBLEM?	☐ Yes	☐ No	☐ NA
16.	Does health worker check for visible SEVERE WASTING?	☐ Yes	☐ No	☐ NA
17.	Does health worker look for PALMAR PALLOR?	☐ Yes	☐ No	☐ NA
18.	Does health worker look for OEDEMA of both feet?	☐ Yes	☐ No	☐ NA
19.	Does health worker check child's weight against a GROWTH CHART?	☐ Yes	☐ No	☐ NA
20.	Does health worker ask whether the child has been given ANTI-HELMINTH?	☐ Yes	☐ No	☐ NA
21.	Does health worker ask about BREASTFEEDING?	☐ Yes	☐ No	☐ NA
22.	Does health worker ask whether the child takes any other FOODS/FLUIDS?	☐ Yes	☐ No	☐ NA
23.	Does health worker ask whether FEEDING CHANGED during illness?	☐ Yes	☐ No	☐ NA
24.	Does the health worker administer or prescribe ORS? (for diarrhoea/dehydration)	☐ Yes	☐ No	☐ NA
25.	Does the health worker provide Zinc tablet? (for diarrhoea)	☐ Yes	☐ No	☐ NA
26.	Does the health worker explain the need to give more liquid or breast milk at home?	☐ Yes	☐ No	☐ NA
27.	Does the health worker explain the need to continue feeding or breastfeeding at home?	☐ Yes	☐ No	☐ NA
28.	Did the health worker advise the caregiver on frequency of feeding?	☐ Yes	☐ No	☐ NA
29.	If yes, what was the advice?<br>*(Multiple response possible)*	Continue breast-feeding .... 1<br>More frequent feeding..................... 2<br>Feed normal amount as before........ 3<br>Other(*specify)*.................................. 99		
30.	Does the health worker tell the caretaker to bring the child back immediately for the danger signs?	☐ Yes	☐ No	☐ NA
31.	Does the health worker use the IMCI chart booklet at any time during the management of the child?	☐ Yes	☐ No	☐ NA
32.	Does health worker use mother's card or any other visual job aids for demonstrating IYCF practice?	☐ Yes	☐ No	☐ NA
33.	Does health worker COUNSEL mothers on food and care required for underweight children?	☐ Yes	☐ No	☐ NA

No	Observation points	Responses
34.	If yes, what did she say?<br>*(Multiple response possible)*	Breastfeed before giving any nutritional food/ supplement .... 1<br>Encourage the child to eat .... 2<br>Offer plenty of breast-milk<br>or clean water .... 3<br>Feed baby with clean cup and spoon4<br>Wash hand before feeding the child 5
35.	Counselled on ensuring for all children aged 6-11 months 1 blue Vitamin A capsule and for all children aged 1-5 years 1 red Vitamin A capsule	☐ Yes ☐ No ☐ NA
36.	Gave age-specific advice on inclusion of salt in all complementary food for children	☐ Yes ☐ No ☐ NA
37.	Use of BCC materials to raise awareness on nutrition	☐ Yes ☐ No ☐ NA
38.	Breastfeeding Demonstration if required (including appropriate position and attachment)	☐ Yes ☐ No ☐ NA
39.	Prescribe/Provide Vitamin-A capsules	☐ Yes ☐ No ☐ NA
40.	Prescribe/Provide Tablet or syrup for deworming	☐ Yes ☐ No ☐ NA

*Please review the entire observation checklist for any **missing points**,*

Fill up with (√) in appropriate box and finish the observation.

International Centre for Diarrhoeal Disease Research, Bangladesh (icddr,b)

Assessing the progress of the National Nutrition Services (NNS) Program

Exit interview Questionnaire for Care giver of sick under-five child

Identification of the health facility

District: ________________|___|___|	Upazila: ____________________|___|___|

Union: ____________________|___|___|	Ward No. |___|

Type of facility
- Upazila Health Complex..1
- Union Health and Family Welfare Centre..............2
- Community Clinic...3

Client information

1.	Code of the respondent অংশগ্রহনকারীর কোডঃ	
2.	Child age শিশুর বয়স	[__][__] months মাস
3.	Sex of the child শিশুর লিঙ্গ	Male পুরুষ .... 1<br>Female মহিলা 2
4.	Child date of birth শিশুর জন্ম তারিখ	[ ][ ] [ ][ ] [ ][ ][ ][ ]<br>Day দিন Month মাস Year বছর
5.	Sex of the caregiver সেবাদানকারীর লিঙ্গ	Male পুরুষ .... 1<br>Female মহিলা 2
6.	Relationship to child: শিশুর সাথে সম্পর্ক	Biological parent জন্মদাতা পিতামাতা .... 1<br>Other relative অন্যান্য আত্মীয় 2

Satisfaction with services সেবায় সন্তুষ্টি

How do you rate the overall service quality at the facility? আপনি কিভাবে কেন্দ্রের সেবার মানকে মূল্যায়ন করবেন?

How satisfied were you with the following: নিচের বিষয়গুলোতে আপনি কতটুকু সন্তুষ্ট ছিলেন		1	2	3	4	5	Code
1.	How is the route or transportation system from your house to this center? আপনার বাড়ি থেকে কেন্দ্রে আসার যানবাহন বা যোগাযোগ ব্যবস্থা কেমন?	Very bad খুবই খারাপ	Bad খারাপ	Average মোটামুটি	Good ভালো	Very Good খুবই ভালো	☐
2.	How long do you have to wait to visit the doctor after you come to this facility? এই কেন্দ্রে আসার পর ডাক্তার দেখাতে আপনাকে কতোক্ষন অপেক্ষা করতে হয়?	Too long অনেক সময়	Somewhat Long কিছুটা বেশি সময়	Neither long nor short খুব বেশি সময়ও না, খুব অল্প সময়ও না	Somewhat short কিছুক্ষন সময়	Very short একেবারেই অল্প সময়	☐
3.	How is the situation of the area where you wait for the doctor? যে জায়গায় আপনি অপেক্ষা করেন তার পরিবেশ কেমন?	Very Bad খুবই খারাপ	Bad খারাপ	Average মোটামুটি	Good ভালো	Very Good খুবই ভালো	☐
4.	How is the advice/prescription of the doctor? ডাক্তার যে পরামর্শ বা প্রেসক্রিপশন দেন তা কেমন?	Very Bad খুবই খারাপ	Bad খারাপ	Average মোটামুটি	Good ভালো	Very Good খুবই ভালো	☐
5.	How much opportunity is there to ask questions to the doctor? ডাক্তারের কাছে প্রশ্ন করার কতোটুকু সুযোগ আছে?	Absolutely no opportunity একেবারেই সুযোগ নেই	Not that much opportunity খুব একটা সুযোগ নেই	Neither much nor less খুব বেশিও না, খুব কমও না	There is some opportunity কিছুটা সুযোগ আছে	There is plenty of opportunity অনেক সুযোগ আছে	☐

Thank you so much for your time and patience with this interview

সাক্ষতকারে সময়দান এবং ধৈর্যধারনের জন্য আপনাকে অসংখ্য ধন্যবাদ।

International Centre for Diarrhoeal Disease Research, Bangladesh (icddr,b)

Assessing the progress of National Nutrition Services (NNS) Program

Service Provider Survey

জেলা/ District/: ___________|___|___| উপজেলাঃ/Upazila: ______________|___|___|

ইউনিয়ন/ Union: ______________________|___|___| ওয়ার্ড নংঃ Ward No. |______|

সেবাকেন্দ্রের নাম/Facility Name/Code____|___|___|

জরিপকারীর নাম এবং আই,ডি Surveyor Name and ID: ___________|____|____| তারিখ/Date: ____/____/____

সেবাকেন্দ্রের ধরণ/ Type of facility	Upazila Health Complex .... 1<br>Union Health and Family Welfare Centre .... 2<br>Community Clinic 3
স্বাস্থ্যসেবাদানকারীর কোডঃ Code of health service provider	|___|___|___|
স্বাস্থ্যসেবাদানকারীর ধরণ/ Designation of health service provider	মেডিকেল অফিসার/ Medical officer .... 1<br>নার্স/ Nurse .... 2<br>পরিবার কল্যান পরিদর্শিকা/ FWV.. 3<br>সাব-এসিস্ট্যান্ট কমিউনিটি মেডিকেল অফিসার/ SACMO .... 4<br>কমিউনিটি স্বাস্থ্যসেবাদানকারী/ CHCP...................................... 5<br>স্বাস্থ্য সহকারী/ HA .... 6<br>পরিবার কল্যাণ সহকারী/ FWA 7

স্বাস্থ্যসেবাদানকারীর প্রাথমিক তথ্যঃ Background information of health service provider

Q#	প্রশ্নসমূহ/ **Questions**	উত্তর/ **Responses**
1.	স্বাস্থ্যসেবাদানকারীর বয়স/<br>Age of health service provider	|___|___| বছর/years
2.	স্বাস্থ্যসেবাদানকারীর লিঙ্গ/<br>Sex of health service provider	পুরুষ/Male..1<br>নারী/ Female..2
3.	স্বাস্থ্যসেবাদানকারী আজকে যেখানে দায়িত্ব পালন করছেন/ Place of service provision on the day of visit	IMCI পুষ্টি কর্নার/ IMCI nutrition corner.............................1<br>প্রসব-পুর্ববর্তী সেবাকক্ষ/ ANC room 2
4.	আপনার সর্বোচ্চ পড়াশোনা কতদূর উল্লেখ করুন/<br>What is the highest level of education you have achieved?	এসএসসি এর নীচে যে ক্লাশ পাশ করেছেন/<br>Completed years of schooling......................... |___|___| এসএসসি/ SSC.. 10<br>এইচএসসি/ HSC.. 12<br>ডিপ্লোমা/Diploma.. 14<br>ব্যাচেলর/ Bachelor .... 15<br>ব্যাচেলর এবং অনার্স/ Bachelor with honors....................... 16<br>মাস্টার্স অথবা উচতর ডিগ্রী/ Masters or Higher 17

স্বাস্থ্য-সেবাদানকারীর প্রশিক্ষণ সংক্রান্ত তথ্য/ Training of health care providers

1.	আপনি কখনো NNS থেকে আয়োজিত পুষ্টি বিষয়ক মৌলিক প্রশিক্ষণ পেয়েছেন?<br>Have you ever received Basic Nutrition Training from NNS?	☐ হ্যা/ Yes ☐ না/ No
2.	আপনি কি কখনো পুষ্টি বিষয়ক যে কোন ধরণের প্রশিক্ষণ পেয়েছেন/<br>Did you ever receive any kind of training on nutrition?	☐ হ্যা/ Yes ☐ না/ No
3.	আপনি কি NNS থেকে IYCF সংক্রান্ত কোন প্রশিক্ষণ পেয়েছেন?<br>Did you receive IYCF training provided by NNS?	☐ হ্যা/ Yes ☐ না/ No
4.	আপনি কি NNS থেকে শিশুর গ্রোথ মনিটরিং সংক্রান্ত কোন প্রশিক্ষণ পেয়েছেন?<br>Did you receive growth monitoring training provided by NNS?	☐ হ্যা/ Yes ☐ না/ No
5.	আপনি কি NNS থেকে মারাত্মক অপুষ্টি এর ব্যবস্থাপনা সংক্রান্ত কোন প্রশিক্ষণ পেয়েছেন?<br>Did you receive training on SAM management provided by NNS?	☐ হ্যা/ Yes ☐ না/ No

গর্ভকালীন পুষ্টি সংক্রান্ত জ্ঞান/ Knowledge about nutrion during pregancy

No.	Question	Code
1.	নবজাতকের খাওয়ানোর ব্যাপারে একজন গর্ভবতী মা'কে কি বিষয়ে ধারণা দেওয়া উচিত?<br>What infant feeding messages should be given to a pregnant woman?<br>(multiple choices)	জন্মের সাথে সাথে বুকের দুধ খাওয়ানো শুরু করার ব্যাপারে/<br>On Early initiation of breastfeeding .... 1<br>৬ মাস পর্যন্ত শুধুমাত্র বুকের দুধ খাওয়ানো সম্পর্কে/<br>On exclusive breast feeding for 6 months....................... 2<br>প্রথম ৬ মাসে দুধ ছাড়া অন্য কিছু খাওয়ানোর কুফল সম্পর্কে/ On dangers of feeding prelacteals and other liquids in the first 6 months .... 3<br>আয়রন-ফলিক এসিড সম্পর্কে / Iron folic acid supplementation..... 4

		জানা নেই/ Don't know.. 8
2.	একজন গর্ভবতি মায়ের কি কি বাড়তি/পরিপূরক পুষ্টি গ্রহণ করা উচিত? What supplements mothers should take during pregnancy? (multiple choices)	আয়রন-ফলিক এসিড/ Iron folic acid... 1<br>ক্যালসিয়াম/ Calcium .... 2<br>ভিটামিন/ Vitamin (Mention:___________________) .... 3<br>পুষ্টি-সম্পুরক প্যাকেট/ Nutrition supplement packet .... 4<br>আয়ডিনযুক্ত লবণ/Iodized Salt.. 5<br>জানা নেই/ Don't know.. 8

শিশুর খাওয়ানো এবং পুষ্টি সংক্রান্ত জ্ঞান/ Knowledge about child feeding and nutrition.

No	Question	Code
1.	জন্মের কত সময় পর বুকের দুধ খাওয়ানো শুরু করা উচিত?<br>How long after birth should a baby start breastfeeding?	জন্মের সাথে সাথেই/ Immediately .... 1<br>জন্মের ১ ঘন্টার মধ্যেই/ within 1 hour.. 2<br>২৪ ঘন্টার মধ্যেই less than 24 hrs .... 3<br>১ দিন পরে/ 1 day later... 4<br>১ দিনেরও বেশী পরে/ More than 1 day later.................................... 5<br>জানা নেই/ Don't know 8
2.	শুধুমাত্র বুকের দুধ খাওয়ানো বলতে কি বোঝায়? What is Exclusive Breastfeeding?	বুকের দুধ ও পানি খাওয়ানো/ Giving baby breast milk and water .....1<br>বুকের দুধ ও অন্যান্য তরল (মধু, চা) Giving baby breast milk and some other liquids (honey, tea…) ....2<br>অন্য কোন তরল-নরম খাওয়া না দিয়ে(পানিও নয়) শুধুমাত্র বুকের দুধ খাওয়ানো/ Giving baby only breast milk and no other liquids or solids, not even water ....3<br>জানা নেই/ Don't know 8
3.	একজন মা যদি মনে করে তার বাচ্চা ঠিকমত বুকের দুধ পাচ্ছেনা, তার কি করা উচিত?<br>If a mother thinks her baby is not getting enough breast milk, what should she do?	বারবার বুকের দুধ খাওয়াবে/ Breastfeed more often/more frequently...............................1<br>অন্যান্য তরল খাওয়াবে/ Give other liquids/foods ....2<br>মায়ের নিজের বেশী পানি খাওয়া উচিত/ Mother needs to drink more water.......................................3<br>মায়ের নিজের বেশী খাবার খাওয়া উচিত/ Mother needs to eat more food ....4<br>মায়ের বেশী বিশ্রাম নেওয়া উচিত/Mother need to rest more ....5<br>বাচ্চাকে ফর্মুলা দুধ খাওয়ানো উচিত Give baby formula..........................6<br>জানা নেই/ Don't know 8
4.	একটি ৪ মাস বয়সের বাচ্চার মা অসুস্থ হয়ে পড়লে তার কি বুকের দুধ খাওয়ানো বন্ধ করা উচিত?<br>Do you think that the mother of a 4-month-old baby should stop breastfeeding if the mother becomes ill?	হ্যা/ Yes ....1<br>না/ No ....2<br>জানা নেই/ Don't know 8
5.	কত বয়স পর্যন্ত বাচ্চাকে বুকের দুধ খাওয়ানো চালিয়ে যাওয়া উচিত?Until about what age should a baby continue to be breastfed?	[___] মাসের সংখ্যা/ Number of months<br>জানা নেই/ Don't know 8

No	Question	Code
6.	কত মাস বয়সে বুকের দুধের সাথে বাচ্চাকে পানি খাওয়ানো শুরু করা উচিত? At what age should a baby first start to receive water along with breast milk?	[___] মাসের সংখ্যা/ Number of months<br>জানা নেই/ Don't know 8
7.	কত মাস বয়সে বুকের দুধের সাথে বাচ্চার প্রথম তরল খাবার শুরু করা উচিত?(যেমন ফরমুলা দুধ, ফলের রস) At what age should a baby first start to receive liquids (including formula, juice) other than breast milk?	[___] মাসের সংখ্যা/ Number of months<br>জানা নেই/ Don't know 8
8.	কত মাস বয়সে বুকের দুধের সাথে বাচ্চার প্রথম নরম খাবার (জাউ ভাত, খিচুড়ি) শুরু করা উচিত?<br>At what age should a baby first start to receive semi-solid (rice, khichuri) food other than breast milk?	[___] মাসের সংখ্যা/ Number of months<br>জানা নেই/ Don't know 8
9.	কত মাস বয়সে বাচ্চার প্রথম প্রানিজ উৎসের (মুরগি, গরুর মাংস, ডিম, মাছ) খাবার শুরু করা উচিত?<br>At what age should a baby start to receive animal source foods (beef, chicken, egg, fish)	[___] মাসের সংখ্যা/ Number of months<br>জানা নেই/ Don't know 8
10.	যে বাচ্চাটি এখনও বুকের দুধ খাচ্ছে, তার দিনে কতবার সম্পুরক/বাড়তি খাবার খাওয়া উচিত?<br>How many times per day should an infant that is still breastfeeding eat? (if don't know, write 98)	**৭ মাস বয়স থেকে ৯মাসের নীচে 7 to <9 months**: প্রধান খাদ্য Main meal [ ]; হাল্কা নাসতা Snack [ ]<br>**৯ মাস বয়স থেকে ১২মাসের নীচে 9 to <12 months**: প্রধান খাদ্য Main meal [ ]; হাল্কা নাসতা Snack [ ]<br>**১২ মাস বয়স থেকে ২৪মাসের নীচে 12 to < 24 months**: প্রধান খাদ্য Main meal [ ]; হাল্কা নাসতা Snack [ ]
11.	একটি শিশুর বৃদ্ধির জন্যে কি কি খাবার খাওয়া উচিত?<br>What foods does a young child need in order to grow?<br>(Multiple choices)	রুটি/ভাত/খিচুরি bread/rice/khichuri/other ....1<br>মাংস/ Meat...2<br>মাছ/ Fish ....3<br>ডিম/ Eggs ....4<br>ফল/ Fruits...5<br>সবজি/ Vegetables...6<br>দুধ Milk ....7<br>তেল/চর্বি Oil/Fat...8<br>জানা নেই/ Don't know 9

No	Question	Code
12	**৬ মাসের নীচের** শিশুর ডায়রিয়া অথবা অন্য কোন ধরণের অসুখ হলে মায়ের কি করা উচিত?<br>What should mother do when the child (below 6 months) has an illness?<br><br>*(Multiple choices)*	ঘরে তৈরী অথবা প্যাকেট স্যালাইন/ Give ORS/home-prepared solution ....1<br>স্বাভাবিকের চেয়ে কম খাওয়াতে হবে/ Feed less than usual ....2<br>স্বাভাবিকের মতই খাওয়াতে হবে/Feed as much food as usual ....3<br>স্বাভাবিকের চেয়ে বেশি খাওয়াতে হবে/Feed more than usual ....4<br>বেশী করে বুকের দুধ খাওয়াতে হবে/Breastfeed more often ....5<br>পুষ্টিকর নরম খাবার খাওয়াবে/ Give nutritious semi/solid food ....6<br>ভেষজ ঔষধ দিতে হবে/ Give traditional medicine.........................7<br>ভাতের মাড় দিতে হবে/ Give rice water..8<br>জানা নেই/ Don't know..9
13	ডায়রিয়ার সময়ে বাচ্চাকে কি বাড়তি কোন পুষ্টি উপাদান দেওয়া প্রয়োজন? হলে সেটা কি? In case of diarrhoea should the child be given any supplement? If yes what?	জিঙ্ক বড়ি/ Zinc tablet .... 1<br>কোন সম্পূরক খাবার প্রয়োজন নেই/No supplement required .... 2<br>জানা নেই/ Don't know... 8

International Centre for Diarrhoeal Disease Research, Bangladesh (icddr,b)

Assessing progress of the National Nutrition Services (NNS) Program

<u>Facility Assessment Checklist</u>

Identification information

District: ___________________|___|___| Upazila: ___________________|___|___|

Union: ___________________|___|___| Ward No. |___|

Facility Name/Code_________________________________|___|___|

Surveyor Name and ID: _____________________________|____|____| Date: ____/____/____

Type of facility	Upazila Health Complex .... 1<br>Union Health and Family Welfare Centre .... 2<br>Community Clinic.. 3
Designation of respondent	Civil surgeon/Superintendent .... 1<br>UHFPO .... 2<br>Medical officer .... 3<br>FWV .... 4<br>SACMO.. 5<br>CHCP .... 6<br>Other(*specify)*______________________________ 9

Availability of logistics and job aids

**Equipment, logistics and job aids**		**Available (Y/N)**	**Functioning (Y/N)**
**ANC/PNC room**			
1.	Accessible and working adult weighing scale		
2.	Accessible and working height scale		
3.	Working blood pressure machine		
4.	Working Stethoscope		
5.	Measuring tape		
6.	Working Thermometer		

Equipment, logistics and job aids		Available (Y/N)	Functioning (Y/N)
7.	Record keeping register		
8.	Pictorial cards with maternal danger signs		
9.	Guideline on Iron Folic Acid/Anaemia prevention and control		
10.	Guideline on distribution of Vitamin-A		
11.	Basic Nutrition Training Guideline (present in the room)		
12.	IYCF manual		
	**IMCI-Nutrition corner**		
13.	Weighing Scale		
14.	Height Scale		
15.	Length Scale		
16.	MUAC Tape		
17.	GMP Card (Girls and Boys)		
18.	Register and reporting Form		
19.	IMCI chart booklet		
20.	Module on basic nutrition (present in the room)		
21.	Guidelines for management of SAM/MAM		
22.	Guideline on Community-based management acute malnutrition		
23.	IYCF manual		
24.	Guideline on distribution of Vitamin-A		
25.	Guideline on distribution of tablets and syrup for deworming		
26.	Iron and folic acid		
27.	Calcium supplements		
28.	Referral slip		
