# Supplementary material for: Quality of nutrition services in primary health care facilities: Implications for integrating nutrition into the health system in Bangladesh
Source: PLoS One. 2017 May 18;12(5):e0178121. doi: 10.1371/journal.pone.0178121 (PMC5436890; doi:10.1371/journal.pone.0178121)
Supplement: S1 Table — (DOCX) [file pone.0178121.s002.docx]

S1 Table: Median consultation time and interquartile range (IQR) of ANC and sick under 5 children within the sub-categories of provider types and their training status

| Provider characteristics | ANC consultation | | Sick under 5 children consultation | |
| --- | --- | --- | --- | --- |
|  | Median time (minutes) | IQR^a^ (minutes) | Median time (minutes) | IQR^a^ (minutes) |
| Total | 5 | 2-60 | 3 | 1-18 |
| Type of providers |  |  |  |  |
| Medical Officer or Nurse | 5 | 3-60 | 3 | 2-6 |
| Family welfare visitor or Sub-assistant community medical officer | 5 | 3-27 | 3 | 1-15 |
| Community healthcare provider or Family welfare assistant | 7 | 5-15 | 3 | 2-10 |
| Training status |  |  |  |  |
| Received basic NNS nutrition training | 5 | 3-17 | 3 | 1-16 |
| Received EmOC training | 9 | 5-12 | - | - |
| Received IMCI training | - | - | 3 | 1-16 |

^a^ IQR= Range between 25% to 75% percentile values of consultation time
