# Supplementary material for: Quality of nutrition services in primary health care facilities: Implications for integrating nutrition into the health system in Bangladesh
Source: PLoS One. 2017 May 18;12(5):e0178121. doi: 10.1371/journal.pone.0178121 (PMC5436890; doi:10.1371/journal.pone.0178121)
Supplement: S2 Table — (DOCX) [file pone.0178121.s003.docx]

S2 Table: Mothers’/caregivers’ level of satisfaction with the nutrition service received for their sick under-five child, n=541

| Client Satisfaction areas | n (%) | | | | | Mean Score | SD |
| --- | --- | --- | --- | --- | --- | --- | --- |
|  | Very poor | Poor | Average | Good | Very good |  |  |
| The ease of travelling to the facility | 17(3.14) | 58(10.7) | 112(20.7) | 303(56.0) | 51(9.43) | 3.6 | 0.9 |
| Waiting time to see the service provider after arriving at the facility | 47(8.7) | 85(15.7) | 122(22.6) | 184(34.0) | 103(19.0) | 3.4 | 1.2 |
| Environment of the waiting area | 11(2.0) | 39(7.2) | 171(31.6) | 299(55.3) | 21(3.9) | 3.5 | 0.8 |
| Satisfaction with the advice/prescription given | 3(0.6) | 4(0.7) | 164(30.3) | 333(61.6) | 37(6.8) | 3.7 | 0.6 |
| Opportunity to ask questions | 26(4.8) | 36(6.7) | 147(27.2) | 226(41.8) | 106(19.6) | 3.6 | 1.0 |
